# Probiotic white cheese production using coculture with *Lactobacillus* species isolated from traditional cheeses

**DOI:** 10.14202/vetworld.2018.726-730

**Published:** 2018-05-30

**Authors:** A. Ehsani, M. Hashemi, A. Afshari, M. Aminzare

**Affiliations:** 1 Department of Food Science and Technology, Faculty of Nutrition, Tabriz University of Medical Sciences, Tabriz, Iran; 2 Department of Nutrition, Faculty of Medicine, Mashhad University of Medical Sciences, Mashhad, Iran; 3 Department of Food Safety and Hygiene, School of Public Health, Zanjan University of Medical Sciences, Zanjan, Iran

**Keywords:** heterofermentative, *Lactobacillus*, probiotic, starter, traditional cheeses

## Abstract

**Aim:**

The aim of the present study was to investigate the viability of lactic acid bacteria isolated from traditional cheeses and cocultured in Iranian white cheese during ripening.

**Materials and Methods:**

A total of 24 samples were isolated from 8 types of traditional cheeses in West Azerbaijan, Iran. Isolated species were cocultured with starter bacteria during the production of Iranian white cheese, and their viability was investigated up to 60 days of the refrigerated storage.

**Results:**

Of 118 isolates of *Lactobacillus*, 73 isolates (62%) were confirmed as facultative heterofermentative and 45 isolates (38%) as obligate homofermentative. Of the facultative heterofermentatives, 28 isolates (24%) were *Lactobacillus plantarum*, 24 isolates (20%) were *Lactobacillus casei*, and 21 isolates (18%) were *Lactobacillus agilis*. Obligate homofermentatives were *Lactobacillus delbrueckii* (21%)*, Lactobacillus helveticus* (14%), and *Lactobacillus salivarius* (3%). *L. plantarum, L. casei* and *L. helveticus* were found in high enough levels(10^6^ CFU/g).

**Conclusion:**

According to the obtained data, it is recommended that complex starters such as *L. helveticus*, *L. plantarum*, and *L. casei* can be used in industrial productions of cheese to obtain exclusive properties of traditional cheeses.

## Introduction

Cheeses made from pasteurized milk have better hygienic quality and uniform texture than traditional ones; however, pasteurization has some devastating effects on the quality of the cheese as it can destroy the flavor-producing microorganisms. To overcome this problem, traditional starters are added after pasteurization [1]. Therefore, knowing the composition of lactic flora, naturally present in traditional cheeses, provides safe and standard starters while maintaining the essential characteristics [2].

Since West Azerbaijan Province (Iran) has environmental and ethnic diversity, there are varieties of traditional foods, particularly dairy products, in this region [3]. There are different kinds of cheese traditionally produced in this region. Urmia Koopeh Cheese is a fermented milk product and one of the most traditional and popular cheese in the West and North-West of Iran, Turkey, and Northern areas of Iraq. It is a semisoft sheep cheese and is produced without using any starter. Another well-known cheese in this area is Lighvan, which is a soft cheese (60% humidity) traditionally produced from raw sheep and goat milk without adding any starter culture [4], the fermentation also depends on the lactic flora of the raw milk. Lighvan cheese is one of the most common dairy products in Iran [5]. Shal cheese, which is also called fresh cheese, is produced traditionally in Urmia in West Azerbaijan province in large casts and has popularity in this region. Salty cheese is another milk product, traditionally made from boiled buttermilk along with salt. In this process, the clot-on-boiling is filtered, and consequently, the result is a salty cheese with particular aroma and flavor [5].

The traditional production of cheese and the replacement of traditional lactic acid bacteria with a variety of non-native microflora are a future possibility in dairy technology; therefore, it would be encouraging for researchers to collect different strains and species of lactic acid bacteria from different dairy products obtained from various regions.

The aim of the current study was to isolate *Lactobacillus* species from different traditional cheeses of West Azerbaijan province, Iran, to produce a white cheese with a uniform texture and a flavor similar to that of traditional cheeses as well as determining starter’s viability in dairy products.

## Materials and Methods

### Ethical approval

This study was approved by the Research Committee of Urmia University, Urmia, Iran.

### Sampling

A total of 24 samples (250 g) were collected from 8 traditional cheeses of different geographical regions in West Azerbaijan (Urmia, Bukan, Mahabad, Sardasht, and Maku), Iran, as follow: Triplicate samples from five different types of Koopeh cheese (15 samples), Shal fresh cheese (3 samples), Lighvan cheese (3 samples), and Salty cheese (3 samples). Samples were kept at 4°C for further analysis.

### Isolation of lactic acid bacteria

Amount of 25 g of each sample was aseptically weighted and homogenized with 225 ml of sodium citrate using a stomacher (Seward Stomacher 400 Circulator, UK). Samples were subsequently diluted (1:10) using sterile peptone water and 0.1 ml from each dilution was sub-cultured on Man Rogosa and Sharpe (MRS) agar (Merck, Germany) used for isolating lactic acid bacteria [6]. Plates were incubated at 30°C and 37°C for mesophilic lactic acid bacteria and at 42°C for thermophilic lactic acid bacteria for 48 h. MRS agar plates were incubated anaerobically using the gas pack systems (Anaerocult C, Merck, Germany). To differentiate homo- and heterofermentative strains, all the isolates were tested for gram reaction, catalase production, and carbohydrates fermentation (homofermentatives showed ribose +, sorbitol +, raffinose ±, lactose +, galactose +, maltose +, melibiose ±, rhamnose −, xylose ±, manose +, salicine +, sorbose +, glucose +, and sucrose + pattern, heterofermentatives showed ribose +, sorbitol ±, raffinose ±, lactose ±, galactose ±, maltose +, melibiose ±, rhamnose ±, xylose −, manose +, salicine ±, sorbose ±, glucose +, and sucrose±pattern). Cimon citrate test was also conducted as a differential test [7,8].

### Iranian white cheese preparation

Starter (Chr. Hansen R 704: *Lactococcus lactis* Subsp., cremoris and *L. lactis* Subsp., diacetyl lactis) and isolated *Lactobacillus* species were added (0.5% w/v and 10^9^ CFU/ml, respectively) to each vat of pasteurized cow milk (65°C for 30 min) separately. A solution of 20 g. 100/L CaCl_2_ was added to each batch, and the fermentation continued for 2-4 h until the pH reached to 6.1-6.2. Then, 0.001% w/v of rennet was added to each vat to coagulate the milk. To improve the efficiency of rennet, the milk temperature was maintained at about 35°C during the formation of cheese clots. After coagulation, the curds were divided into small cubes (1-2 cm^3^) and pressed. Cheese samples were allowed to rest in 20% w/v brine salt for 8 h and in 8% sterile brine-salt for 15 days at 12-14°C. Final ripening applied for 45 days at 4°C [9]. The manufacture of Iranian white cheese was performed in triplicate for each treatment. *Lactobacillus* count was performed 1 h after inoculation and on days 3, 7, 15, 30, 45, and 60.

### Lactobacillus viable cell counts

For viability assessment of probiotic bacteria during 60 days of storage at refrigerator temperature, 10 g of cheeses were homogenized with 90 mL of sterile sodium citrate using a stomacher. Decimal dilutions in peptone water were prepared and plated on MRS agar (Merck, Germany). Plates were incubated at 37°C for 48 h [9]. Finally, colonies were Gram stained, and catalase production, carbohydrates fermentation, and Cimon citrate test were performed.

## Results

A total of 118 *Lactobacillus* isolates were identified from 4 different kinds of cheese. Phenotypic characterization of isolates, such as gram reaction, catalase production, and carbohydrates fermentation (Table-1), resulted in the identification of 73 isolates (62%) of *Lactobacillus* as facultative heterofermentative and 45 isolates (38%) as obligate homofermentative.

**Table-1:** Results of Gram staining and biochemical tests of *Lactobacillus* species.

Tests	Bacterial strain					

	1	2	3	4	5	6
Gram staining	+	+	+	+	+	+
Ribose	+	+	+	+	+	+
Sorbitol	+	+	+	−	+	+
Raffinose	+	−	+	−	−	+
Lactose	+	+	+	−	+	+
Galactose	+	+	+	+	−	+
Maltose	+	+	+	+	+	+
Melibiose	+	−	−	+	−	+
Rhamnose	−	−	−	+	−	−
Xylose	−	−	+	−	−	−
Manose	+	+	+	+	+	+
Salicine	+	+	+	−	+	+
Sorbose	+	+	+	+	−	+
Glucose	+	+	+	+	+	+
Sucrose	+	+	+	−	+	+
CO_2_ production from glucose	+	−	+	−	−	+
Growth at 30°	+	+	+	−	−	+
Growth at 37°	+	+	−	+	−	+
Growth at 42°	−	−	−	+	+	−
Catalase	−	−	−	−	−	−
Citrate	+	−	−	+	−	+

1=*Lactobacillus plantarum,* 2=*Lactobacillus casei,* 3=*Lactobacillus* *agilis,* 4=*Lactobacillus helveticus,* 5=*Lactobacillus delbrueckii*, 6=*Lactobacillus salivarius*

**Figure-1:**
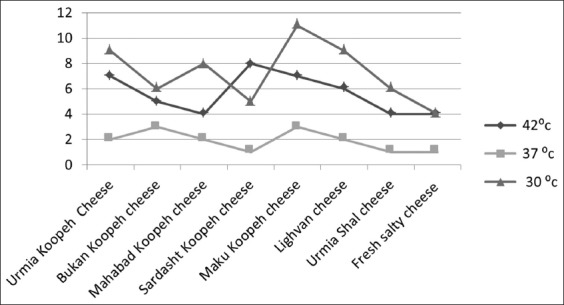
Lactobacillus isolates in various traditional cheeses at different temperatures.

Facultative heterofermentatives were composed of 28 isolates (24%) of *Lactobacillus plantarum*, 24 isolates (20%) of *Lactobacillus Casei*, and 21 isolates (18%) of *Lactobacillus agilis*. Obligate homofermentative *Lactobacillus* species were composed of 25 isolates (21%) of *Lactobacillus delbrueckii*, 16 isolates (14%) of *Lactobacillus helveticus*, and 4 isolates (3%) of *Lactobacillus salivarius*. 58 *Lactobacillus* isolates (49%) were identified at 30°C, 15 isolates (13%) at 37°C, and 45 isolates (38%) at 42°C. Distribution of *Lactobacillus* isolates in traditional cheeses is shown in Figure-1.

*Lactobacillus* viability of probiotic white cheese samples during 60 days of storage is presented in Table-2. The viability of *L. helveticus*, *L. plantarum*, and *L. casei* was observed above 6 Log CFU/g, throughout 60 days of storage period. *L. plantarum* and *L. casei* were different from *L. helveticus*, and the differences were statistically significant (p<0.05). Other species declined below 6 log CFU/g after 30 days of storage (Table-2).

**Table-2:** *Lactobacillus* viability of probiotic white cheese samples during storage time (mean values±SD).

*Lactobacillus* species	*Lactobacillus* count (Log_10_ CFU/g)						

	Time (days)						

	0	3	7	15	30	45	60
*L. plantarum*	9.81±0.03^Aa^	8.96±0.01^Ab^	7.95±0.00^Ac^	7.74±0.04^Ad^	6.72±0.04^Ae^	6.63±0.06^Aef^	6.52±0.07^Af^
*L. casei*	9.73±0.1^Aa^	8.92±0.01^ABb^	7.79±0.04^Bc^	6.94±0.02^Bd^	6.61±0.12^Ae^	6.36±0.1^Bf^	6.50±0.04^Aef^
*L. agilis*	9.82±0.05^Aa^	8.84±0.00^BCb^	7.60±0.00^Bc^	6.52±0.07^Cd^	5.91±0.01^Be^	5.72±0.04^Cf^	5.52±0.07^Bg^
*L. helveticus*	9.32±0.12^Ba^	8.81±0.03^Cb^	8.07±0.22^Ac^	7.78±0.01^Ac^	6.61±0.02^Ade^	6.36±±0.10^Be^	6.72±0.04^Ad^
*L. delbrueckii*	9.44±0.12^Ba^	8.52±0.07^Db^	7.87±0.05^ABc^	6.82±0.04^Bd^	4.36±0.10^Ce^	4.67±0.05^Df^	4.56±0.07^Ce^
*L. salivarius*	9.77±0.07^Aa^	9.48±0.03^Eb^	7.62±0.03^Bc^	5.79±0.08^Dd^	4.98±0.03^De^	4.94±0.03^Ee^	4.72±0.04^Ce^

Same small letters within the same row indicate non-significant differences (p>0.05) based on Tukey’s Range Test. Same capital letters within the same column indicate non-significant differences (p>0.05) based on Tukey’s range test, SD=Standard deviation, *L. plantarum=Lactobacillus plantarum, L. casei=Lactobacillus casei, L. agilis=Lactobacillus agilis, L. helveticus=Lactobacillus helveticus, L. delbrueckii=Lactobacillus delbrueckii, L. salivarius=Lactobacillus salivarius*

## Discussion

Knowing the microbial population of traditional dairy products results in better understanding of specific characteristics of such products [10]. The technology used in the different geographical area in producing traditional dairy products has a direct impact on the diversity of the microbial population [10,11]. *Lactobacillus* species are identified as dominant species in cheeses produced from raw milk because they can grow under severe selective conditions and cause desirable sensory properties due to their proteolytic activities [12]. In the current study, mesophilic strains of *Lactobacillus* were dominant lactic flora of traditional cheeses since 62% of *Lactobacillus* strains were isolated at 30°C and 37°C. In a study by Ahmadi *et al*. [13], on lactic acid bacteria of Lighvan cheese, mesophilic (85%, 46 isolates) and thermophilic (15%, 8 isolates) *Lactobacillus* were isolated, respectively. Previous studies indicated that facultative heterofermentative *Lactobacillus*, especially *L*. *plantarum* and *L. casei*, is the most typical isolated lactobacilli from cows and sheep milk [14], which can also produce specific flavor and aroma [15]. In a previous study on Fossa cheese, it was determined that *L*. *plantarum*, *L*. *paracasei*, and *L*. *carvatus* produce small peptides and volatile amino acids during activities of dipeptidase, aminopeptidase, endopeptidase, and proteinase enzymes, leading to the production of desirable flavor and aroma [16]. In other studies, these bacteria were identified as dominant species of Iranian Lighvan cheese, South African traditional fermented milk and Maasai traditional fermented milk (Kenya) which are all consistent with the results of this study [17-19]. These results confirm the important role of lactic acid bacteria isolated from traditional cheeses.

In this study, *L*. *plantarum* was the dominant *Lactobacilli* of traditional cheeses, especially Urmia Koopeh cheese. Of 118 isolated *Lactobacillus* strains, 28 isolates (24%) were *L*. *plantarum*. These mesophilic bacteria are able to fermentate citrate and proteolysis during cheese curing [20]. Results of similar studies have shown such differences in the microbial composition of fermented foods, using culture-dependent and independent methods [21].

Lactic acid bacteria are responsible for potential usefulness of fermented milk products [22]. Many researchers have studied the antibacterial activity of lactic acid bacteria and their ability to produce antimicrobial substances and destroying pathogens [23,24].

In recent years, various preservatives including natural additives have been introduced to overcome pathogenic microorganisms [25,26]. The effect of lactic acid bacteria in preventing the growth of pathogens plays an important role in reducing the use of chemical additives and preservatives. It also improves consumer’s satisfaction by obtaining healthier and ready-to-use foods [27]. There have been several studies on the enrichment of fermented milk products with probiotics, such as enrichment of Cheddar [28], Manchego [29], Cottage [30], Turkish Beyaz [31], Argentinean [32], and white cheese [33].

Among probiotic bacteria, *L*. *plantarum, L. casei*, and *L. helveticus* were present in high enough levels (10^6^ CFU/g threshold) throughout the storage, which is required for probiotic activity and having satisfactory viability (count decreases during 60 days <3 log order) in cheese. In the current study, *L*. *agilis*, *L*. *delbrueckii*, and *L. salivarius* reduced at least 4 logs during 60 days of the storage and reached to the final count of <5.52±0.07 log CFU/g. The adverse conditions such as low pH, lack of carbohydrates, and probably unfavorable ambient temperature during 60 days of the storage might be responsible for this reduction [34]. This reduction in *Lactobacillus* population, constructed various strategies such as microencapsulation with different materials to protect microorganisms for the gradual release of microorganism in cheese [35].

## Conclusion

Results of this study indicated that bacterial isolates obtained from traditional cheeses could be used in industrial cheese production to obtain exclusive properties of traditional cheeses. The outcome of the study also showed that *L. plantarum, L. casei*, and *L. helveticus* are good combinations of starter cultures with acceptable viability.

## Authors’ Contributions

AE supervised the project. MH carried out the experiment. AA wrote the manuscript with support from Dr. Ehsani and Hashemi. MA contributed to the final version of the manuscript. All authors read and approved the final manuscript.
